# A step-by-step method for cultural annotation by LLMs

**DOI:** 10.3389/frai.2024.1365508

**Published:** 2024-05-01

**Authors:** Edgar Dubourg, Valentin Thouzeau, Nicolas Baumard

**Affiliations:** Département d’études cognitives, Institut Jean Nicod, École normale supérieure, Université PSL, Paris, France

**Keywords:** automatic annotation, human cultures, large language models, annotation loop, tutorial

## Abstract

Building on the growing body of research highlighting the capabilities of Large Language Models (LLMs) like Generative Pre-trained Transformers (GPT), this paper presents a structured pipeline for the annotation of cultural (big) data through such LLMs, offering a detailed methodology for leveraging GPT’s computational abilities. Our approach provides researchers across various fields with a method for efficient and scalable analysis of cultural phenomena, showcasing the potential of LLMs in the empirical study of human cultures. LLMs proficiency in processing and interpreting complex data finds relevance in tasks such as annotating descriptions of non-industrial societies, measuring the importance of specific themes in stories, or evaluating psychological constructs in texts across societies or historical periods. These applications demonstrate the model’s versatility in serving disciplines like cultural anthropology, cultural psychology, cultural history, and cultural sciences at large.

## 1 Introduction

The study of human cultures has always presented a formidable challenge to researchers aiming for a scientific and empirical approach (e.g., Gottschall, 2008; Moretti, 2014). This challenge arises from the volume and diversity of data that needs to be handled, processed, and analyzed consistently. This has become even more evident since the emergence of the digital age (Acerbi, 2020). Cultural data is often vast, but also scattered and heterogeneous, making it a daunting task to gather and interpret it meaningfully.

Historically, the computational humanities have employed several methods for large-scale data collection and analysis. However, these methodologies have been shown to be inherently limited. For instance, participatory manual collection involves direct data gathering from individuals through surveys and observations, but it is considered costly, time-consuming, and limited in scale (Wang et al., 2021). Another approach is interrogating pre-existing databases which may contain historical records and artifact descriptions, such as IMDb for movies (e.g., Sreenivasan, 2013; Canet, 2016; Dubourg et al., 2023) or HRAF for anthropological texts (e.g., Garfield et al., 2016; Boyer, 2020; Singh, 2021). While these databases offer a wealth of information, they often lack uniformity and consistency, making it difficult to use them to compare different cultures and time periods. For instance, we cannot straightforwardly use the Science Fiction tag of Wikipedia to track the evolution of the number of Science Fiction works over time, as this category emerged quite late in history, even though it may be applicable to earlier works. Additionally, methods like embedding and bag-of-words have been used to analyze textual descriptions, converting text into numerical vectors for large dataset analysis (e.g., Martins and Baumard, 2020). Despite their utility, these computational techniques are hard to implement and sometimes fall short in capturing the contextual understanding necessary for classifying or rating cultural items.

In line with many other researchers, we believe that recent advancements in language models pre-trained on massive amounts of textual information have heralded a new era in the scientific study of culture (see Bail, 2023, for a review; see Binz and Schulz, 2023). These LLMs, like Generative Pre-trained Transformers (GPT), appear for the first time to be capable of annotating and parsing cultural data on a vast scale, and at an abstract level, by leveraging the contextual understanding inherent in these models (Brown et al., 2020; Grossmann et al., 2023). Unlike static online databases, GPT cross-references and synthesizes a wide range of texts, creating uniform annotations by linking information not explicitly connected in its training data. While LLMs definitively cannot replace human expertise in all aspects of scientific fields interested in human culture, they can replace cultural data annotation tasks, with multiple advantages for research, including: (1) Cost-effectiveness—GPT can annotate tens of thousands of cultural descriptions within a few hours. (2) Uniformity—GPT facilitates a consistent annotation process capable of handling descriptions or titles across various historical periods, societies, and media types. (3) Objectivity—compared to human annotations for similar tasks, GPT’s approach is less dependent upon idiosyncrasies (see Kjeldgaard-Christiansen, 2024; see Section 3).

This paper primarily serves as a practical guide for their application. We introduce a detailed, step-by-step pipeline for utilizing LLMs in empirical studies. This guide is designed to provide practical insights for various applications of such automatic annotation, including annotating Human Relations Area Files (HRAF) descriptive accounts of non-industrial societies, generating or annotating descriptions of cultural items such as novels, video games or technological patents, analyzing folklore narratives, or extracting thematic elements from human-generated texts. Note that we strongly advocate for pairing LLM methods with other more established research techniques in all studies where it is possible, enabling case-by-case convergence testing and facilitating future meta-analyses. In all, this paper does not seek to debate whether LLMs can (Abdurahman et al., 2023) or should (Crockett and Messeri, 2023) be used in science (see also Brinkmann et al., 2023); instead, it provides a concrete how-to guide for applying these models to large cultural datasets.

## 2 Method for automatic annotation

**Figure 1:**
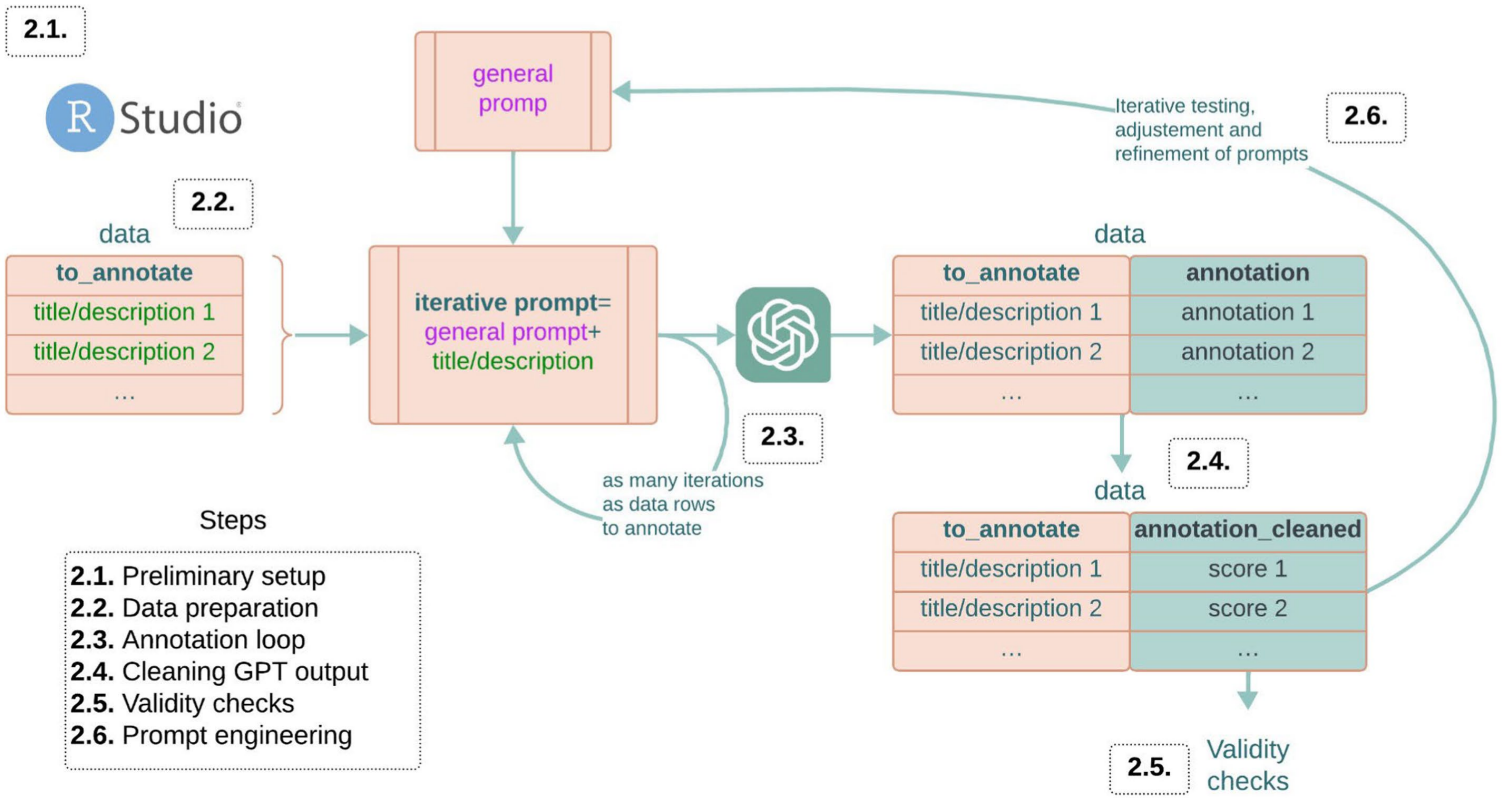
Schematic representation of all the steps for the cultural data annotation process.

Throughout this guide, specific R code snippets are included in the text. These can be directly copied and pasted into your R programming console. This script can be downloaded in an R format here: https://osf.io/3q6zb/. Alongside the code, we provide explanations for each step and decision in the methodology. For those who prefer to see an application of the methodology before diving into the details, refer to Section 2.7. This section presents a practical example that demonstrates how the methodology can be applied to a real-world research project. It serves as a reference point to contextualize the steps discussed throughout the tutorial (see Figure 1).

### 2.1 Preliminary setup

In this methodology, we have selected R and GPT-4 based on their widespread use in relevant fields (see https://rstudio-education.github.io/hopr/starting.html to install R and RStudio). However, note that this approach can be adapted to other programming languages and LLMs. Python is a suitable alternative for R, and models like Bard or LLaMA can be used instead of GPT-4 (with some adjustments in the code). These alternatives can be chosen based on the researcher’s familiarity and the specific requirements of their project. To start, the ‘httr’ and ‘tidyverse’ packages in R are essential for interfacing with web APIs, particularly for accessing the OpenAI platform and GPT-4. Install this package using the following code:
             install.packages(‘httr’)
             library(httr)
             install.packages(‘tidyverse’)
             library(tidyverse)


In addition to the programming setup, a premium OpenAI account is required to access GPT’s capabilities via its API. This account provides an API key, a unique identifier necessary to authenticate and make requests to the OpenAI services. To obtain this key, log in to your OpenAI account, navigate to the ‘Account’ section, and then to the ‘Keys’ subsection. Here, you can find or generate your API key. This key will be crucial in the subsequent steps of the methodology, as it allows your R scripts to communicate with the GPT-4 model and send data for annotation. To effectively manage your usage and track expenses associated with using the OpenAI API, you can monitor the ‘Usage’ section in your OpenAI account. As of the latest information (December 2023), using GPT-3.5-turbo is priced at $0.0080 per 1,000 tokens (a token being roughly equivalent to a word). To annotate 50 book summaries of 500 tokens, it would therefore cost $0.20. Using the updated pricing for GPT-4 Turbo, which offers an 8 k context length, annotating 50 book summaries, each 500 tokens long, totals $1.50 at $0.06 per 1,000 output tokens.

### 2.2 Data preparation

The dataset that is going to be annotated could encompass anything from novel titles or video game titles to more detailed texts like summaries of books or descriptions of social practices. Let us note that there are two distinct approaches to using GPT for data annotation, depending on the nature of your dataset:

*Title-based knowledge retrieval*: In cases where the dataset consists of brief information like titles of well-known cultural artifacts, GPT can leverage its vast pre-existing knowledge. For example, when given just the title of a video game, GPT can use its comprehensive database to provide accurate ratings or categorizations (e.g., Dubourg and Chambon, 2023). This approach relies on the model’s ability to tap into a wealth of accumulated information about widely recognized items (see Chang et al., 2023, for a study about books known by GPT-4).
*Textual annotation*: This approach applies when the dataset includes more detailed textual content, such as plot summaries or descriptions of artifacts. For example, if the dataset contains descriptive accounts of hunter-gatherer social behaviors, you can use GPT to assess specific cultural aspects, like the presence of third-party punishment. This method relies on GPT’s ability to interpret and analyze the given text.

It is crucial to understand that even in the Textual Annotation approach, GPT’s vast knowledge plays a significant role. For instance, if you prompt GPT with a movie plot summary asking for an evaluation of a certain aspect (like the importance of the theme of love), GPT is likely to identify the movie from the summary and assess the requested feature based on its extensive understanding of the movie, beyond just the plot details provided. Therefore, the distinction between title-based knowledge retrieval and textual annotation is only significant in the extent to which it guides how you structure your dataset, how you design your prompts, and how you test the validity of the annotation (e.g., title-based knowledge retrieval relies entirely on GPT’s internal knowledge, necessitating careful verification). However, the overall process of interfacing with GPT remains consistent regardless of the approach.

Your final dataset (let us call it data) should have a column called to_annotate which will be taken as input in the GPT prompt. It could therefore be a string of titles or of detailed textual information.

### 2.3 Annotation loop

The annotation process using GPT-4 is implemented through a script. Here’s a breakdown of the script’s steps with portions of the code included:

Start by setting up your OpenAI API key for authentication. Place your API key in the script:
             my_API <- ‘PUT YOUR API HERE’

Then, create a function named hey_chatGPT to handle sending prompts to GPT and receiving responses (adapted from: https://rpubs.com/nirmal/setting_chat_gpt_R). The function is structured to make POST requests to the OpenAI API, using your API key for authentication. The hey_chatGPT function includes error handling and retry logic to ensure reliable communication with the API. If a request fails or an error occurs, the function retries up to a maximum number of times, defined in the script (here, 3).

            hey_chatGPT <- function(prompt) {
              retries <- 0
              max_retries <- 3  # Set a maximum number of retries
              while (retries < max_retries) {
                tryCatch({
                  chat_GPT_answer <- POST(
                    url = "
https://api.openai.com/v1/chat/completions",
                    add_headers(Authorization = paste("Bearer", my_API)),
                    content_type_json(),
                    encode = "json",
                    body = list(
                      model = "PUT A GPT MODEL HERE",
                      temperature = 0,
                      messages = list(
                        list(role = "system", content = "PUT THE ROLE OF GPT HERE"),
                        list(role = "user", content = prompt)
                      )
                    )
                  )

                  if (status_code(chat_GPT_answer) != 200) {
                    print(paste("API request failed with status", status_code(chat_GPT_answer)))
                    retries <- retries + 1
                    Sys.sleep(1)  # Wait a second before retrying
                  } else {
                    result <- content(chat_GPT_answer)$choices[[1]]$message$content
                    if (nchar(result) > 0) {
                      return(str_trim(result))
                    } else {
                      print("Received empty result, retrying...")
                      retries <- retries + 1
                      Sys.sleep(1)  # Wait a second before retrying
                    }
                  }
                }, error = function(e) {
                  print(paste("Error occurred:", e))
                  retries <- retries + 1
                  Sys.sleep(1)  # Wait a second before retrying
                })
              }
              return(NA)  # Return NA if all retries failed
            }



Note that in the function, two important parameters need to be customized according to your specific research needs. First, within the function, there is a placeholder for specifying which GPT model you intend to use, indicated by “PUT A GPT MODEL HERE.” GPT-4, being the most advanced and efficient model available, is typically the preferred choice for difficult tasks. However, it is important to note that GPT-4 might also incur higher costs. Depending on your project’s requirements and budget, you might opt for other models like GPT-3.5 or earlier versions. Replace the placeholder with the model ID of your chosen GPT version (models’ name to find here: https://platform.openai.com/docs/models). Second, the role parameter, indicated by “PUT THE ROLE OF GPT HERE,” is crucial in guiding the kind of responses you expect from GPT. This role defines the nature of GPT’s interaction in the conversation. For instance, if your project involves analyzing films, you might define GPT’s role as a “film expert.” This role setting helps in aligning GPT’s responses with the specific perspective or context required for your research. Modify this parameter to reflect the role that best fits the context of your project.

After customizing these parameters, prepare the dataset for annotation by creating a new column to hold GPT’s annotations. Assign a placeholder (NA) to each entry in this new column:

             data$annotation <- NA

Then, the following script runs a loop over each row of the to_annotate column in your dataset. In each iteration, it constructs a prompt by combining a predefined question or statement with the specific data point and then sends this concatenated prompt to GPT:

             for (i in 1:nrow(data)) {
               prompt <- "PUT YOUR PROMPT HERE"
               to_annotate <- data$to_annotate[i]
               concat <- paste(prompt, to_annotate)
               result <- hey_chatGPT(concat)
               data$annotation[i] <- result
               }



As each prompt is processed by GPT, the response is captured and stored in the designated annotation column. The loop prints the response for monitoring.

Note that, in the script, the “PUT YOUR PROMPT HERE” placeholder within the loop is where you need to insert the specific prompt that will guide GPT’s annotation process. This prompt should be crafted to elicit the desired information from GPT (see Section 2.7).

When constructing the prompt for each data point, it is important to frame it based on the type of annotation approach you are using. For title-based knowledge retrieval, the prompt should end with “The title is:.” This prefix indicates to GPT-4 that what follows is a title, and it should tap into its vast knowledge base for annotation. For textual annotation, the prompt should end with “The description is:.” This tells GPT-4 that it will be analyzing a more detailed textual description. The content of the to_annotate column, either titles or textual descriptions, follows this initial prompt, in an iterative manner.

In your prompt construction, also consider the type of output you require from GPT, which can be broadly categorized into two approaches:

*Categorical approach*: Here, you ask GPT to classify each item into one of several predefined categories. For example, in speculative genre labeling of movies, the prompt could be: “Assign each movie to either Fantasy or Science Fiction.” This approach is useful for sorting items into distinct groups or labels.
*Dimensional approach*: Alternatively, you might ask GPT to rate an item on a particular dimension, providing a numerical score. For instance, you could ask: “On a scale from 0 to 10, rate the importance of the imaginary world in the story.” If a numerical score is requested, it is helpful to specify in the prompt that the response should end with a digit, such as: “The end of your response should be \Score = \, with a digit between 0 and 10, with no text, letters, or symbols after.” This specification aids in the extraction of numerical data for analysis (see Section 2.3).

To enhance the usefulness and interpretability of GPT’s responses, especially in the dimensional approach, you can prompt GPT to provide a brief justification before the numerical value. This not only gives context to GPT (leading to more accurate ratings) but also allows for a qualitative assessment of GPT’s reasoning process. For example, “Explain briefly why you assign this score, followed by the score itself, which should be a single digit between 0 and 10.” Prompting GPT to first analyze and then rate encourages its neural network to deeply process the context before quantifying, utilizing the layered design for more context-informed ratings.

Finally, to prevent data inaccuracies and avoid GPT ‘hallucinating’ responses when lacking information (in the textual annotation approach) or knowledge (in the knowledge retrieval approach), include in the prompt: “If unable to annotate due to insufficient data or unrecognized titles, respond with ‘NA’.” This ensures more reliable annotations.

Incorporating these considerations in prompt engineering will help tailor GPT’s output to your specific research needs. This whole script is therefore designed to facilitate the efficient use of GPT-4 for annotating a wide range of textual data. It is particularly suitable for researchers in cultural studies looking to leverage AI for large-scale data analysis.

### 2.4 Cleaning output

After running the annotation loop, it is often necessary to clean the output from GPT, especially if it includes numerical annotations like scores or binary classifications (i.e., with the dimensional approach). This step ensures that the data is in a usable format for analysis. The script uses a regular expression pattern to identify and extract numerical values from GPT’s output. The pattern is designed to recognize numbers (single or double digits) that appear at the end of a sentence or before a space. This is crucial when GPT outputs a mix of text and numbers, and you only need the numerical value. After identifying the numerical patterns in GPT’s responses, the script extracts these numbers and converts them into a numeric format.
             pattern <- "(\\b\\d{1,2}\\b)(\\.\\s*|$)"
          data$annotation_cleaned <- as.numeric(
                as.integer(str_extract(data$annotation, pattern)))



This cleaning process is especially important in studies where quantitative measures are derived from qualitative data, such as rating scales or classifications.

### 2.5 Validity check

#### 2.5.1 Internal validity check

Internal validity, which refers to the consistency of the results within the scope of the study, can be checked to establish the reliability of the study’s outcomes. To assess the internal validity, the main method involves using multiple annotations with different prompts. This technique allows for checking the inter-rater agreement between various iterations of GPT annotations on the same data. For instance, annotating the same set of cultural artifacts with slightly varied prompts and comparing the consistency of GPT-4’s responses. Let us note that, in the hey_chatGPT function, the temperature parameter is set to 0. A temperature of 0 means that GPT will generate the most likely response, reducing randomness, and therefore enhancing the reproducibility of the output. This setting is especially important when aiming for consistent and precise annotations across multiple runs. Note that increasing the temperature can be useful in tasks where creativity or diversity in responses is desired.

#### 2.5.2 External validity check

In the context of Automatic Annotation, external validity assesses whether the LLM’s annotations accurately reflect real-world phenomena. This is important because we aim to ensure that GPT’s interpretations or classifications are not just internally consistent but also truly representative of the cultural artifacts or behaviors they are annotating. The methods to check the external validity include:

*Random sampling for qualitative evaluation*: A straightforward method is to manually review a random sample of GPT’s annotations. This review can confirm whether the annotations align with the actual content or nature of the data points.
*Statistical comparison with manual annotation*: For a random subsample, compare GPT’s annotations with those made by human annotators. This involves statistical analysis to see how closely GPT’s ratings or categorizations match with those done manually. A high degree of correlation would indicate good external validity.
*Statistical comparison with expected metadata*: This involves checking GPT’s annotations against existing relevant metadata. For example, in annotating movies for the presence of love, one would expect these annotations to correlate with the ‘Romance’ tag in IMDb metadata. However, perfect correlation is not always expected. Here, genres are broad categories and may not precisely capture all content nuances (e.g., a movie can include a love story without being tagged as a Romance). A statistically significant association, nonetheless, would suggest that GPT’s annotations are valid in reflecting real-world characteristics.
*Statistical comparison with other methods*: Other computational linguistic techniques can be used, such as word frequency analysis, semantic embeddings, and topic modeling, to independently assess cultural data.
*Expert consultation*: Engaging experts in the annotation process can provide insights into the nuances that LLMs might overlook or misinterpret. For instance, when annotating cultural artifacts or practices from various societies, experts can help ensure that the annotations respect the subtleties of those cultures.

To enhance the credibility and reproducibility of research, it is highly recommended to make the full prompts and outputs used in the study transparent. This can be achieved by including detailed appendices in publications or making the data available in public repositories.

### 2.6 Prompt engineering

Prompt engineering is a crucial preliminary step in the process of using LLMs like GPT for automatic annotation. It involves crafting the queries or instructions (prompts) that are fed to the model to elicit the most accurate and relevant responses. This step is essential because the quality and specificity of the prompts significantly influence the model’s output. Notably, Liu et al. (2022) revealed that through optimized prompt-tuning results akin to fine-tuning can be achieved. Unlike fine-tuning, which requires retraining the model on a specific dataset to adjust its parameters (and therefore demands high levels of computational resources and programming skills), prompt engineering simply involves formulating effective prompts that direct the existing model’s capabilities. This emphasizes the potential of prompt engineering as a viable alternative to more resource-intensive model training methods. This process should be undertaken before initiating the annotation loop (Section 2.3) to ensure the model is properly guided to provide the desired output.

The method outlined below for prompt engineering is not the only or definitive approach; rather, it serves as a guiding framework. Researchers should feel encouraged to adapt and evolve this process based on their specific project needs.

*Trial and error with multiple prompts*: Experiment with various prompts on GPT’s playground (https://platform.openai.com/playground) or within ChatGPT (noting that ChatGPT does not allow for temperature adjustments). The goal is to find prompts that consistently yield coherent and relevant outputs.
*Adjustment of prompts*: If a response from GPT seems off, ask the model to explain its rating or annotation, providing insights into its reasoning process. For further refinement, initiate a different discussion where you present GPT with the prompt, the content to be annotated, and the model’s initial response. Explicitly point out inaccuracies or issues in the response and then ask GPT how it would reconstruct the prompt to avoid such errors. This iterative process helps in fine-tuning the prompts.
*Iterative testing and refinement*: Repeat this process for about 20 cultural artifacts or descriptions that are well-known to the researchers. This familiarity allows for a better assessment of GPT’s responses. This iterative approach helps in identifying the most effective prompt structure for your specific annotation task.

After testing multiple prompts, you can conduct a qualitative analysis of the outputs. Review the responses to determine which prompts consistently produced the most accurate and relevant annotations. Select the prompt or prompts that work best for your dataset and research objectives. Prompt engineering is a dynamic and iterative process that requires experimentation. The time invested in this stage can significantly enhance the quality of the data annotation process, leading to more reliable research findings.

### 2.7 Example

To illustrate a practical application of the methodology outlined in the previous sections, we present an example from a recent study that utilized GPT for automatically annotating video game titles based on specific dimensions. This study, conducted by Dubourg and Chambon (2023), aimed to score video games on the dimensions of the DEEP model–Discovering, Experimenting, Expanding, and Performing.

The dataset comprised titles of 16,000 video games, which were to be annotated along the DEEP dimensions, with a title-based knowledge retrieval approach. The prompts were theory-driven, reflecting each of the DEEP dimensions. For instance, for the Discovering dimension, the prompt was:

Discovering is about using novel and innovative actions or strategies to achieve abstract goals. Discovering involves actively exploring the game world, uncovering hidden secrets, and engaging in non-linear gameplay elements. It includes the ability to undertake side quests or optional objectives that offer new insights, items, or areas to explore. You will rate a video game on a scale of 0 to 100 on this dimension. This score will reflect how well the game aligns with the characteristics and potential of this dimension. Give a single number, without text. The video game is:

This prompt encapsulates the theoretical underpinnings of the dimension and is used to instruct GPT to tap into its extensive knowledge base for rating.

The same R script and annotation loop described in Section 2.3 were employed. The script was designed to prompt GPT-3.5 with each video game title and the relevant DEEP dimension, receive the model’s rating, and then store this rating in the dataset (for a total cost of less than $200 for 16,000 video games annotated along 4 dimensions). The output from GPT-3.5 was cleaned and processed as described in Section 2.4. This step ensured that the ratings were in a numerical format suitable for analysis.

Here is, as a second example, a potential prompt to measure love in movies:

Rate the significance of love in each movie on a scale from 0 to 10, where 0 represents a complete absence or irrelevance of love, and 10 indicates that love is central and extremely relevant. Focus exclusively on the presence and impact of love between partners, setting aside other forms of love or relationships such as familial bonds or friendships. Provide a very brief justification. You must write the score after / SCORE = / at the end, with no text nor symbol after. If you don’t know the movie, score NA. The movie is:

### 2.8 Further developments

#### 2.8.1 Confidence intervals

Evaluating GPT’s confidence in its annotations can provide additional insight into the reliability of the output. Understanding the model’s self-assessed certainty can help gauge the robustness of the annotations and identify areas where GPT might be less reliable. Here are some methods for assessing GPT’s confidence:

*Evaluating prior knowledge in title-based knowledge retrieval*: In cases where titles are used, ask GPT to rate its knowledge about the title on a scale from 0 to 10 before providing the annotation. This can be done using a prompt like: “On a scale from 0 to 10, how familiar are you with this work. Please provide a number representing your level of knowledge. The work is:” Consider using only responses where GPT rates its knowledge level as 7 or higher for more reliable annotations, for instance.
*Rating confidence in annotations*: Regardless of the approach (title-based retrieval or textual approach), after receiving an annotation, you can prompt GPT to rate its confidence in that annotation. For instance: “Rate your confidence in your previous answer on a scale from 0 to 10.” Select annotations where GPT’s self-rated confidence is high for increased reliability.
*Direct confidence interval in dimensional approach*: In scenarios where GPT is asked to provide a score on a dimension, you can also ask for a confidence interval along with the score. A prompt might include: “Provide a score between 0 and 10 for the following aspect, and also give a confidence interval for your score.” The confidence interval can be a range (e.g., 5–7), indicating the range within which GPT believes the true score lies. This method adds a layer of probabilistic assessment to the annotations, giving a sense of the range of uncertainty in GPT’s response.

Incorporating confidence evaluations in the annotation process adds reliability to the analysis, allowing researchers to distinguish between more and less certain annotations. This approach can refine the data selection process, leading to a more nuanced understanding of the model’s capabilities and limitations.

#### 2.8.2 Chains of thoughts

Chain-of-thought prompting is a technique that has the potential to enhance the precision of annotations in complex tasks (Wei et al., 2023). This method involves prompting language models like GPT to decompose tasks into intermediate steps, providing a detailed breakdown of the reasoning process. This approach not only improves the accuracy of the model’s responses in complex reasoning scenarios but also offers interpretability by revealing how conclusions are reached. It is particularly beneficial in cultural or social science research where the data often involves multiple layers of interpretation. For instance, in a study analyzing the cultural significance of ritual practices in various societies, chain-of-thought prompting could be used to ask GPT to first identify key elements of a ritual described in a text, then analyze their symbolic meanings, and finally assign them a numerical rating on some relevant dimensions. By using chain-of-thought prompting, researchers can more reliably interpret the model’s analysis, making it a valuable tool for analysis in cultural studies.

## 3 Advantages and limits of automatic cultural annotation

### 3.1 Cost-effectiveness: the capabilities of LLMs at zero-shot knowledge intensive tasks

It has been argued that the ability to generate domain-specific and structured data rapidly, as well as convert structured knowledge into natural sentences, opens new possibilities for data collection (Ding et al., 2023; see Abdurahman et al., 2023, for a review). The use of GPT-3 for data annotation has shown promising results, with its accuracy and intercoder agreement surpassing those of human annotators in many Natural-Language Processing (NLP) tasks (Wang et al., 2021; Gilardi et al., 2023; Rathje et al., 2023; Webb et al., 2023). Even more crucially, GPT is now adept at *zero-shot learning* applications: it performs well without any re-training of the model (see Qin et al., 2023, for an evaluation of the capacity of ChatGPT to zero-shot learning in 20 different NLP tasks; see Kuzman et al., 2023, on a genre identification task; see also Pei et al., 2023, for a one-shot tuning phase approach).

But what about tasks that require extensive background knowledge? The quality of cultural data annotation is capital, and it often depends on the expertise of the annotators. Pre-trained transformers seem capable of handling even that. For instance, GPT-3 has been employed to automatically generate textual information sheets for artworks, displaying excellent knowledge of art concepts and specific paintings (Bongini et al., 2023). Crucially, this study concludes that there is no need to retrain the model to incorporate new knowledge about the cultural artifacts: “this is possible thanks to the memorization capabilities of GPT-3, which at training time has observed millions of tokens regarding domain-specific knowledge” (Bongini et al., 2023). Let us take an example from another domain of human cultures: GPT-4 performed comparably to well-trained law student annotators in analyzing legal texts, demonstrating again its potential in tasks requiring highly specialized domain expertise (Savelka, 2023; Savelka et al., 2023; see also, Fink et al., 2023, for knowledge extraction from lung cancer report; see Hou and Ji, 2023, for cell type annotation for RNA-seq analysis; see Kjell et al., 2023, for mental health assessment; see Dillion et al., 2023, for moral judgements). In all, GPT appears excellent at zero-shot *knowledge intensive* tasks (see Yang et al., 2023, for a review). These models therefore offer a highly efficient and cost-effective method for cultural data annotation.

### 3.2 Uniformity in annotation: facilitating comparative approaches

There are specific tasks within related fields where LLMs can be particularly beneficial, especially when objective properties need to be extracted from large volumes of data. For instance, quantifying the presence of a theme such as love in a story is a task where objectivity is key. Humans making such assessments may be influenced by their personal interests in romantic love, leading to variability in interpretations. In contrast, LLMs can offer more objective measurements. For scientific studies of human cultures, another crucial aspect is the consistent handling of data from diverse societies, languages, or historical periods. This uniform approach is essential in ensuring analysis of various cultural practices and artifacts, enabling cross-cultural comparisons. In their extensive study across 15 datasets, including 31,789 manually annotated tweets and news headlines, Rathje et al. (2023) tested GPT-3.5 and GPT-4’s ability to accurately detect psychological constructs like sentiment, discrete emotions, and offensiveness across 12 languages, including Turkish, Indonesian, and eight African languages such as Swahili and Amharic. They found that GPT outperformed traditional dictionary-based text analysis methods in these tasks. It highlights GPT’s capability to provide more consistent and accurate cross-cultural comparisons.

The advent of advanced language models like GPT-4 also presents opportunities for comparative analysis across various media forms. Traditional approaches have struggled to directly compare cultural artifacts like movies, video games, TV series, and novels, due to their distinct genres and sensory modalities—visual and auditory in films and games, versus imaginative in literature. However, LLMs enable a more abstract level of analysis, focusing on underlying themes and psychological constructs. By processing and interpreting data at this higher level of abstraction, LLMs can identify and compare core thematic elements, such as love or imaginary worlds, regardless of the medium. It should open new avenues in psychological research studying cultural trends. For instance, an uptick in horror fiction across movies, video games, and novels may not occur uniformly due to each medium’s ability to elicit fear. By analyzing these diverse expressions of a genre like horror throughout all cultural productions, LLMs can provide a comprehensive view of the overall appeal and evolution of recreational horror (see Clasen, 2017; Yang and Zhang, 2022).

### 3.3 Annotating fuzzy concepts and loose categories: facilitating the dimensional approach

One difficulty with cultural annotation is that many concepts used in social and cultural sciences are often difficult to express clearly: What is “agency” exactly (Haggard and Chambon, 2012; Chambon et al., 2014)? How do you define “puritanism” (Fitouchi et al., 2023)? What about “fictionality” (Nielsen et al., 2015; Paige, 2020)? These concepts are fuzzy, and the categories are loose. Characters can be more or less agentic, stories can be more or less fictional, and there are different ways to define puritanism. In fact, scientists often use scales, based on multiple questions. This means that it would be hard to ask a human annotator to rate cultural items on fictionality for instance using a single definition. In these cases, LLM can offer invaluable help. For instance, one can give a LLM the questionnaire used by psychologists to measure agency (Vallacher and Wegner, 1989), and prompt the LLM to rate the level of agency of characters using this questionnaire. GPT will estimate the intensity of the semantic association between the questions and the words associated with the character in a story. LLMs thus enable systematic data collection on complex and hard-to-define aspects of cultural artifacts, fostering the extraction of novel insights (e.g., Piper and Toubia, 2023).

We posit that scientific approaches to human cultures can gain from leveraging LLMs for one last key reason: the facilitation of a dimensional approach (see Section 2.3). The standard approach in cultural sciences has often been categorical. For instance, historians have long debated whether romantic love is a Western medieval invention (De Rougemont, 2016 [1939]), and whether a similar phenomenon is present or not outside the West (Goody, 1998; Pan, 2015). Yet, the aspects we seek to analyze, such as psychological constructs in texts or thematic elements in stories, often are not binary but exist on a continuum. These elements vary in degrees, be it in sensitivity, intensity, prevalence, or importance. Adopting a dimensional approach is more fruitful (Trull and Durrett, 2005). Unlike the categorical approach, which determines whether an individual possesses a trait or not, the dimensional approach used in personality psychology, psychiatry or behavioral ecology uses a continuum to determine the various levels of a trait that an individual may possess. In this perspective, the right question is not whether people in the medieval period fell in love or not but rather quantifying and comparing the intensity of this feeling to other periods (Baumard et al., 2022, 2023).

The move from categorical to dimensional analysis represents a shift in accurately measuring and understanding cultural phenomena. Recognizing this continuous nature is crucial, as it aligns more closely with the real-world diversity of cultural artifacts or social behaviors. In personality psychology, a similar transition occurred: from rigid categorical classifications to a dimensional approach with traits, which better captured the variability of people’s patterns of thoughts and behaviors (e.g., Costa and McCrae, 1992; Kashdan et al., 2018; Dubois et al., 2020). This change was largely enabled by methodological advancements like the emergence of Likert scales and the elaboration of factor analysis. Similarly, in cultural and social sciences, technologies like GPT arguably offer the potential to move beyond categorical thinking. For instance, elements such as love, conflict, or adventure, typically associated with specific genres in movies or literary works, can actually be present to varying degrees across a broad spectrum of works. GPT’s capacity to quantitatively analyze these elements on a scale allows for capturing their presence in a more nuanced manner.

## 4 Conclusion

The ability of GPT to process vast amounts of data with nuanced understanding can show potential in tasks ranging from annotating descriptions of non-industrial societies to extracting psychological constructs from texts, thereby serving a wide array of disciplines including anthropology, psychology, and history. This methodology can also foster interdisciplinary connections. For example, understanding cultural artifacts as cognitive fossils—physical imprints of the psychological traits of their creators or consumers—can bridge gaps between cultural, historical, and psychological sciences (Baumard et al., 2023). The use of LLMs in Automatic Annotation of cultural data can contribute to this interdisciplinary bridge. By enabling the homogenization and comparison of diverse cultural data, LLMs provide a unified methodological ground for multiple research areas interested in human cultures.

Before concluding, it is important to stress again that, for now, we believe that the use of LLMs for cultural data annotation should not be seen as a standalone solution, especially in fields where operationalization and quantification is hard. Rather, it should be used with other traditional research methods or subjected to rigorous validity checks, both internal and external. Multiple studies have highlighted that LLMs can exhibit biases, often reflecting limitations in representing the diversity of personalities, opinions, beliefs, etc. (Santurkar et al., 2023; see Abdurahman et al., 2023, for a review). These biases stem from the data on which these models are trained, potentially impacting their ability to fully grasp and represent the vast spectrum of human experiences and perspectives. Thus, Automatic Cultural Annotation does not advocate for the complete replacement of human judgment in psychological and cultural studies with LLMs.

## Data availability statement

The original contributions presented in the study are included in the article/supplementary material, further inquiries can be directed to the corresponding author.

## Author contributions

ED: Methodology, Project administration, Visualization, Writing – original draft, Writing – review & editing. VT: Methodology, Writing – original draft, Writing – review & editing. NB: Conceptualization, Writing – original draft, Writing – review & editing.

## References

[ref1] Abdurahman S. Atari M. Karimi-Malekabadi F. Xue M. J. Trager J. Park P. S. . (2023). Perils and opportunities in using large language models in psychological research. OSF Preprints. doi: 10.31219/osf.io/tg79n PMC11249969 39015547

[ref2] Acerbi A. (2020). Cultural evolution in the digital age (first edition). Oxford: Oxford University Press.

[ref3] Bail C. A. (2023). Can generative AI improve social science? Soc ArXiv. doi: 10.31235/osf.io/rwtzs PMC11127003 38722813

[ref4] Baumard N. Huillery E. Hyafil A. Safra L. (2022). The cultural evolution of love in literary history. Nat. Hum. Behav. 6, 506–522. doi: 10.1038/s41562-022-01292-z 35256800

[ref5] Baumard N. Safra L. Martins M. D. J. D. Chevallier C. (2023). Cognitive fossils: using cultural artifacts to reconstruct psychological changes throughout history. Trends Cogn. Sci. 28, 172–186. doi: 10.1016/j.tics.2023.10.001 37949792

[ref6] Binz M. Schulz E. (2023). Using cognitive psychology to understand GPT-3. Proc. Natl. Acad. Sci. USA 120 Scopus:e2218523120. doi: 10.1073/pnas.2218523120, PMID: 36730192 PMC9963545

[ref7] Bongini P. Becattini F. Del Bimbo A. (2023). “Is GPT-3 all you need for visual question answering in cultural heritage?” in Computer vision – ECCV 2022 workshops. eds. Karlinsky L. Michaeli T. Nishino K., vol. 13801 (Switzerland: Springer Nature Switzerland), 268–281.

[ref8] Boyer P. (2020). Informal religious activity outside hegemonic religions: wild traditions and their relevance to evolutionary models. Relig. Brain Behav. 10, 459–472. doi: 10.1080/2153599X.2019.1678518

[ref9] Brinkmann L. Baumann F. Bonnefon J.-F. Derex M. Müller T. F. Nussberger A.-M. . (2023). Machine culture. Nat. Hum. Behav. 7, 1855–1868. doi: 10.1038/s41562-023-01742-2, PMID: 37985914

[ref10] Brown T. B. Mann B. Ryder N. Subbiah M. Kaplan J. Dhariwal P. . (2020). Language models are few-shot learners (arXiv:2005.14165). arXiv. doi: 10.48550/arXiv.2005.14165

[ref11] Canet F. (2016). Quantitative approaches for evaluating the influence of films using the IMDb database. Commun. Soc. 29, 151–172. doi: 10.15581/003.29.2.151-172

[ref12] Chambon V. Sidarus N. Haggard P. (2014). From action intentions to action effects: how does the sense of agency come about? Front. Hum. Neurosci. 8. doi: 10.3389/fnhum.2014.00320, PMID: 24860486 PMC4030148

[ref13] Chang K. K. Cramer M. Soni S. Bamman D. (2023). Speak, memory: an archaeology of books known to ChatGPT/GPT-4 (arXiv:2305.00118). arXiv. doi: 10.48550/arXiv.2305.00118

[ref14] Clasen M. (2017). Why horror seduces. Oxford: Oxford University Press.

[ref15] Costa P. T. McCrae R. R. (1992). Four ways five factors are basic. Personal. Individ. Differ. 13, 653–665. doi: 10.1016/0191-8869(92)90236-I

[ref16] Crockett M. Messeri L. (2023). Should large language models replace human participants? [preprint]. PsyArXiv. doi: 10.31234/osf.io/4zdx9

[ref17] De Rougemont D. 2016 (1939). L’amour et l’Occident, 10–18.

[ref18] Dillion D. Tandon N. Gu Y. Gray K. (2023). Can AI language models replace human participants? Trends Cogn. Sci. 27, 597–600. doi: 10.1016/j.tics.2023.04.008, PMID: 37173156

[ref19] Ding B. Qin C. Liu L. Chia Y. K. Joty S. Li B. . (2023). Is GPT-3 a good data annotator? (arXiv:2212.10450). arXiv. doi: 10.48550/arXiv.2212.10450

[ref9004] Dubourg E. Chambon V. (2023). DEEP: A model of gaming preferences informed by the hierarchical nature of goal-oriented cognition. In Review.

[ref20] Dubois J. Eberhardt F. Paul L. K. Adolphs R. (2020). Personality beyond taxonomy. Nat. Hum. Behav. 4, 1110–1117. doi: 10.1038/s41562-020-00989-3 33173199

[ref9001] Dubourg E. Thouzeau V. de Dampierre C. Mogoutov A. Baumard N. (2023). Exploratory preferences explain the human fascination for imaginary worlds. Scientific Reports, 13. doi: 10.31234/osf.io/d9uqs PMC10225465 37246187

[ref9002] Fitouchi L. André J.-B. Baumard N. (2023). Moral disciplining: The cognitive and evolutionary foundations of puritanical morality. Behavioral and Brain Sciences. Available at: https://osf.io/2stcv. 10.1017/S0140525X22002047 36111617

[ref24] Fink M. A. Bischoff A. Fink C. A. Moll M. Kroschke J. Dulz L. . (2023). Potential of ChatGPT and GPT-4 for data mining of free-text CT reports on lung cancer. Radiology 308:e231362. doi: 10.1148/radiol.231362, PMID: 37724963

[ref25] Garfield Z. H. Garfield M. J. Hewlett B. S. (2016). “A cross-cultural analysis of hunter-gatherer social learning” in Social learning and innovation in contemporary hunter-gatherers: Evolutionary and ethnographic perspectives. eds. Terashima H. Hewlett B. S. (Japan: Springer Japan), 19–34.

[ref26] Gilardi F. Alizadeh M. Kubli M. (2023). ChatGPT outperforms crowd workers for text-annotation tasks. Proc. Natl. Acad. Sci. 120:e2305016120. doi: 10.1073/pnas.2305016120, PMID: 37463210 PMC10372638

[ref27] Goody J. (1998). Food and love: A cultural history of east and west Verso.

[ref28] Gottschall J. (2008). “On method” in Literature, science, and a new humanities (Springer, New York: Springer).

[ref29] Grossmann I. Feinberg M. Parker D. C. Christakis N. A. Tetlock P. E. Cunningham W. A. (2023). AI and the transformation of social science research. Science 380, 1108–1109. doi: 10.1126/science.adi1778 37319216

[ref30] Haggard P. Chambon V. (2012). Sense of agency. Curr. Biol. 22, R390–R392. doi: 10.1016/j.cub.2012.02.040 22625851

[ref31] Hou W. Ji Z. (2023). Reference-free and cost-effective automated cell type annotation with GPT-4 in single-cell RNA-seq analysis.

[ref32] Kashdan T. B. Stiksma M. C. Disabato D. J. McKnight P. E. Bekier J. Kaji J. . (2018). The five-dimensional curiosity scale: capturing the bandwidth of curiosity and identifying four unique subgroups of curious people. J. Res. Pers. 73, 130–149. doi: 10.1016/j.jrp.2017.11.011

[ref33] Kjeldgaard-Christiansen J. (2024). What science can’t know: on scientific objectivity and the human subject. Poetics Today 45, 1–16. doi: 10.1215/03335372-10938579

[ref34] Kjell O. N. E. Kjell K. Schwartz H. A. (2023). Beyond rating scales: with care for targeted validation large language models are poised for psychological assessment [preprint]. PsyArXiv. doi: 10.31234/osf.io/yfd8g PMC11911012 38290286

[ref35] Kuzman T. Mozetič I. Ljubešić N. (2023). ChatGPT: beginning of an end of manual linguistic data annotation? Use case of automatic genre identification (arXiv:2303.03953). arXiv. doi: 10.48550/arXiv.2303.03953

[ref36] Liu X. Ji K. Fu Y. Tam W. L. Du Z. Yang Z. . (2022). P-tuning v2: prompt tuning can be comparable to fine-tuning universally across scales and tasks (arXiv:2110.07602). arXiv. doi: 10.48550/arXiv.2110.07602

[ref9003] Martins M. de J. D. Baumard N. (2020). The rise of prosociality in fiction preceded democratic revolutions in Early Modern Europe. Proceedings of the National Academy of Sciences. 117:202009571. doi: 10.1073/pnas.2009571117 PMC7682547 33127754

[ref37] Moretti F. (2014). “Operationalizing”: Or, the function of measurement in modern literary theory. J. Engl. Lang. Lit. 60, 3–19. doi: 10.15794/JELL.2014.60.1.001

[ref38] Nielsen H. S. Phelan J. Walsh R. (2015). Ten theses about fictionality. Narrative 23, 61–73. doi: 10.1353/nar.2015.0005

[ref39] Paige N. D. (2020). Technologies of the novel. Cambridge: Cambridge University Press.

[ref40] Pan L. (2015). When true love came to China. Hong Kong: Hong Kong Univ. Press.

[ref41] Pei X. Li Y. Xu C. (2023). GPT self-supervision for a better data annotator (arXiv:2306.04349). arXiv. doi: 10.48550/arXiv.2306.04349

[ref42] Piper A. Toubia O. (2023). A quantitative study of non-linearity in storytelling. Poetics 98:101793. doi: 10.1016/j.poetic.2023.101793

[ref43] Qin C. Zhang A. Zhang Z. Chen J. Yasunaga M. Yang D. (2023). Is ChatGPT a general-purpose natural language processing task solver? (arXiv:2302.06476). arXiv. doi: 10.48550/arXiv.2302.06476

[ref44] Rathje S. Mirea D.-M. Sucholutsky I. Marjieh R. Robertson C. Van Bavel J. J. (2023). GPT is an effective tool for multilingual psychological text analysis [preprint]. PsyArXiv. doi: 10.31234/osf.io/sekf5 PMC11348013 39133853

[ref45] Santurkar S. Durmus E. Ladhak F. Lee C. Liang P. Hashimoto T. (2023). Whose opinions do language models reflect? (arXiv:2303.17548) Proceedings of the 40th International Conference on Machine Learning, 202, 29971-30004. arXiv. doi: 10.48550/arXiv.2303.17548

[ref46] Savelka J. (2023). Unlocking practical applications in legal domain: evaluation of GPT for zero-shot semantic annotation of legal texts. In: Proceedings of the Nineteenth International Conference on Artificial Intelligence and Law, pp. 447–451. doi: 10.1145/3594536.3595161

[ref47] Savelka J. Ashley K. D. Gray M. A. Westermann H. Xu H. (2023). Can GPT-4 support analysis of textual data in tasks requiring highly specialized domain expertise? In: Proceedings of the 2023 Conference on Innovation and Technology in Computer Science Education, Vol. 1, pp. 117–123. doi: 10.1145/3587102.3588792

[ref48] Singh M. (2021). Magic, explanations, and evil: on the origins and design of witches and sorcerers. Curr. Anthropol. 62, 2–29. doi: 10.31235/osf.io/pbwc7

[ref49] Sreenivasan S. (2013). Quantitative analysis of the evolution of novelty in cinema through crowdsourced keywords. Sci. Rep. 3:2758. doi: 10.1038/srep02758, PMID: 24067890 PMC3783974

[ref51] Trull T. J. Durrett C. A. (2005). Categorical and dimensional models of personality disorder. Annu. Rev. Clin. Psychol. 1, 355–380. doi: 10.1146/annurev.clinpsy.1.102803.144009 17716092

[ref52] Vallacher R. R. Wegner D. M. (1989). Levels of personal agency: individual variation in action identification. J. Pers. Soc. Psychol. 57, 660–671. doi: 10.1037/0022-3514.57.4.660 2926623

[ref53] Wang S. Liu Y. Xu Y. Zhu C. Zeng M. (2021). Want to reduce labeling cost? GPT-3 can help (arXiv:2108.13487). arXiv. doi: 10.48550/arXiv.2108.13487

[ref54] Webb T. Holyoak K. J. Lu H. (2023). Emergent analogical reasoning in large language models (arXiv:2212.09196). arXiv. doi: 10.48550/arXiv.2212.09196 37524930

[ref55] Wei J. Wang X. Schuurmans D. Bosma M. Ichter B. Xia F. . (2023). Chain-of-thought prompting elicits reasoning in large language models. Advances in neural information processing systems, 2022.

[ref56] Yang J. Jin H. Tang R. Han X. Feng Q. Jiang H. . (2023). Harnessing the power of LLMs in practice: a survey on ChatGPT and beyond (arXiv:2304.13712). arXiv. doi: 10.48550/arXiv.2304.13712

[ref57] Yang H. Zhang K. (2022). How resource scarcity influences the preference for counterhedonic consumption. J. Consum. Res. 48, 904–919. doi: 10.1093/jcr/ucab024

